# Effects of Quenching Cooling Rate on Residual Stress and Mechanical Properties of a Rare-Earth Wrought Magnesium Alloy

**DOI:** 10.3390/ma15165627

**Published:** 2022-08-16

**Authors:** Qiumin Xie, Yunxin Wu, Tao Zhang, Shunli Peng, Zhongyu Yuan

**Affiliations:** 1 School of Mechanical and Electrical Engineering, Central South University, Changsha 410083, China; 2 College of Intelligent Manufacturing and Mechanical Engineering, Hunan Institute of Technology, Hengyang 421002, China; 3 State Key Laboratory of High-Performance Complex Manufacturing, Central South University, Changsha 410083, China; 4 Light Alloy Research Institute, Central South University, Changsha 410083, China

**Keywords:** rare-earth wrought magnesium alloy, quenching rate, microstructure, residual stress, mechanical properties

## Abstract

To investigate the effect of quenching rate on microstructure, residual stress (RS) and mechanical properties of a rare-earth wrought magnesium alloy Mg-Gd-Y-Zr-Ag-Er, RS in 20 °C water quenching (WQ (20 °C)), 100 °C water quenching (WQ (100 °C)) or air cooling (AC) conditions were measured and compared with the simulation results, corresponding mechanical properties and microstructure in quenching and aging state were studied. The decrease of quenching rate has little effect on the grain size but makes the twinning disappear, precipitates increase and the texture weakened, leading to easier brittle fracture after aging. WQ (100 °C) is the best quenching condition in this study, with a significant decline in RS and only 4.9% and 3.7% decrease in yield stress (YS) and hardness compared with WQ (20 °C). The results make it feasible to invent an appropriate quenching method of greatly reducing RS while maintaining mechanical properties. The research conclusions would be beneficial to the application of the alloy.

## 1. Introduction

Magnesium alloys are characterized by low density and high specific strength [1,2], which have broad application prospects in aerospace, automobile, 3C and other fields. However, the deformation ability is inadequate at room temperature due to the lack of slip systems. In recent years, the addition of rare-earth (RE) elements significantly improves the heat resistance, ductility, creep resistance and specific strength of magnesium alloys even at high temperature by precipitation and hardening [3], grain sizes refining [4] and texture weakening [5], which notably extend the application range.

Solution treatment followed by quenching, plus artificial aging, are commonly applied to alloys to enhance the mechanical properties. In the processes, alloy components are dissolved into the alloy matrix to form a supersaturated solid and this state is maintained to room temperature [6], while the following artificial aging causes solute atoms to precipitate in the form of the second phase, and the pinning effect of the second phase hinders dislocation and grain boundary movement [7]. During quenching and cooling, on the other hand, the large temperature gradient brings about inhomogeneous plastic deformation and induces residual stress (RS), which reduces mechanical properties [8], dimensional stability [9], fatigue properties [10] and corrosion resistance [11]. Thus, quenching has both advantages and disadvantages to material properties, and the quenching rate is the key factor to mediate the contradiction between mechanical properties and RS [12].

In the aerospace manufacturing industry, the quenching RS should be reduced to the minimum to prevent excessive deformation in subsequent mechanical processing due to the release of RS, which will affect the accuracy of the final product and even cause a scrap of parts. Moreover, magnesium alloy is easy to crack during quenching compared with steel and aluminum alloy, while quenching combined with aging treatment can effectively improve the mechanical properties; therefore, it is very important to research the variation of RS under different quenching conditions and investigate the mechanical properties simultaneously. RS reduction and mechanical properties enhancement after heat treatment had been studied for years. Gao [13] applied thermal vibratory stress relief (TVSR) method to evidently reduce RS, Robinson [14] employed natural aging and cold compression for stress relieving, Dong [15] proposed rotating backward extrusion (RBE) technique to produce high performance AZ80 alloy cylindrical tubes by decreasing grain size and weakening texture and Zhou [16] applied hot isostatic pressing (HIP) to enhance the integrity and reliability of GW63 alloy. Asl [17] focuses on the effect of deep cryogenic treatment (−196 °C) on microstructure and mechanical properties of AZ91 magnesium alloy. Either reducing RS or enhancing mechanical properties have been studied; however, both aspects should be taken into account as a whole in practice.

The aims of the research are to investigate the effect of quenching rate on microstructure, RS and mechanical properties of a newly developed high-strength heat resistant rare-earth wrought magnesium alloy Mg-Gd-Y-Zr-Ag-Er, and to explore whether it is possible to reduce RS while maintaining mechanical properties. Experiments of solution treatment prior to 20 °C water quenching (WQ (20 °C)), 100 °C water quenching (WQ (100 °C)) or air cooling (AC) were carried out, and with artificial aging thereafter. The effect of quenching rate on microstructure, RS and mechanical properties were manifested. The results would be beneficial to the application of the alloy.

## 2. Experimental

### 2.1. Heat Treatment and Mechanical Properties Measurements

**Figure 1:**
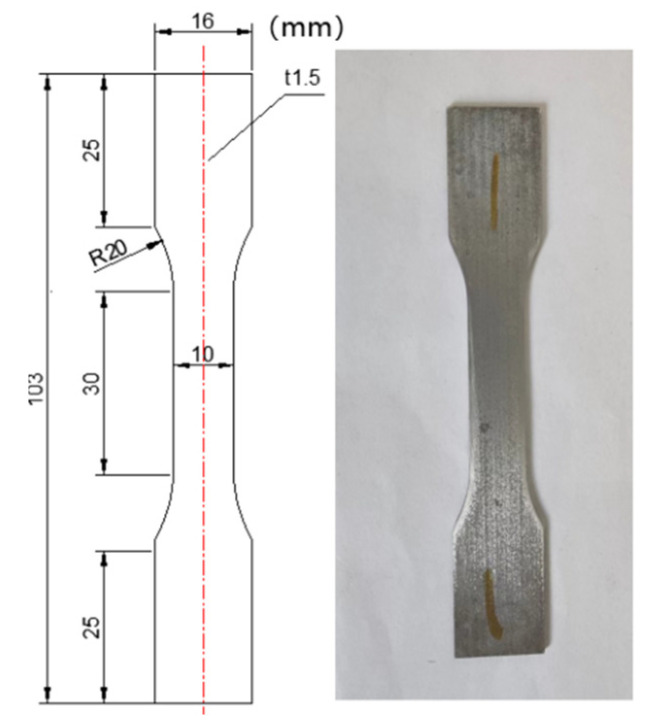
Size of the tensile specimen.

The dimension of the as-received extrusion huge sheet was 1300 mm × 300 mm × 20 mm, three samples in size of 110 mm × 110 mm × 18 mm in extrusion direction (ED), long transverse direction (LTD) and short transverse direction (STD) were taken out from the sheet by using a wire cut electric discharge machine (EDM). Solution treatment at 500 °C for 8 h was conducted in a resistance furnace with a fan, followed by WQ (20 °C), WQ (100 °C) or AC, the transfer time was less than 10 s. After employing wire EDM to split the specimens in half, the cross-sections were utilized to calculate RS using the contour method, which would be covered in Section 2.2. The solution-treated sample was cut into 10 mm × 10 mm × 4 mm cubes and aged at 225 °C for 24 h to attain peak hardness, both solution-treated and aged cubes were measured with a load of 300 g and a dwell time of 15 s, and five points were measured for each specimen. Tensile specimens were also cut by wire EDM from solution-treated and aged samples. The size of the sample is shown in Figure 1, and a tensile test was conducted on the INSTRON-3386 material test machine at a crosshead speed of 1 mm/min at room temperature.

### 2.2. Residual Stress Measurement Methods

**Figure 2:**
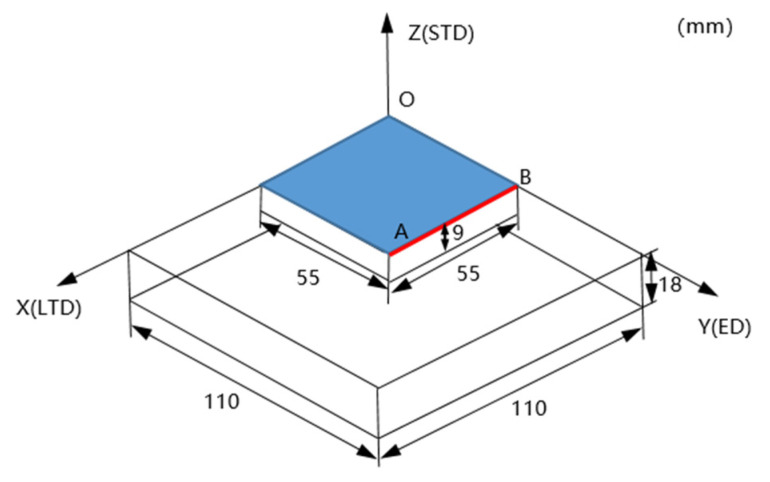
A schematic showing the dimension of the sample for quenching treatment (white) and simulation (blue) and RS measurement path (red line).

As shown in Figure 2, after quenching, considering symmetry, the measurement methods were used to test the RS on the surface of the sample along the direction from point A to point B on the red line, with a total of four points at a constant distance.

**Figure 3:**
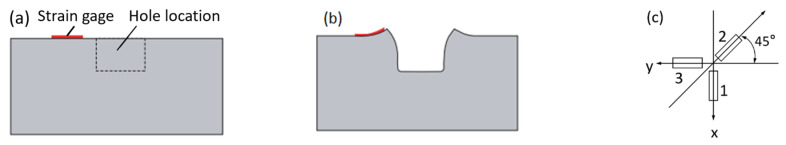
Schematic cross-sections around a hole drilled into tensile RS [19] (**a**) before and (**b**) after hole drilling (**c**) strain gage arrangement direction.

**Figure 4:**
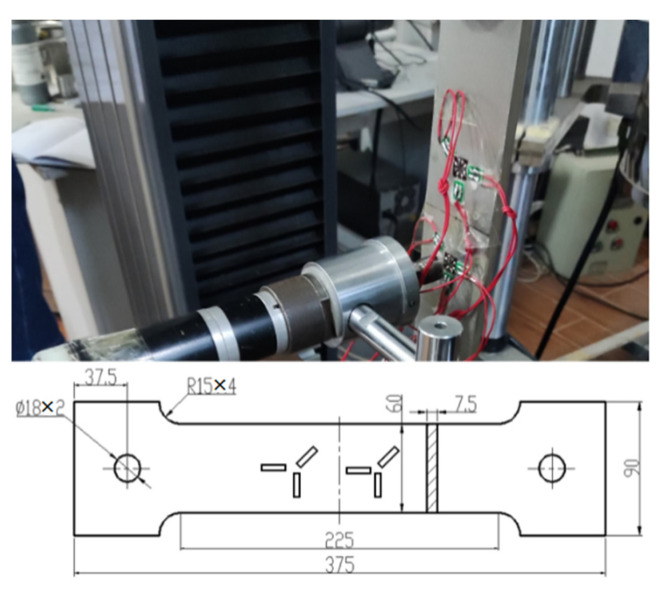
Calibration samples of calibration coefficients A and B.

Both the hole drilling method and the X-ray diffraction (XRD) method were applied to the surface of the samples. The hole drilling method is to drill a small hole in the sample which causes RS to release, as illustrated in Figure 3, with the strain gage around the hole to monitor the value of the strain, and then convert it to the value of RS through Equations (1) and (2) [18].

(1) $$\sigma_{1}=\frac{A+B}{4AB}\left(\Delta \varepsilon_{1}+\frac{B-A}{A+B}\Delta \varepsilon_{3}\right)$$

(2) $$\sigma_{2}=\frac{A+B}{4AB}\left(\Delta \varepsilon_{3}+\frac{B-A}{A+B}\Delta \varepsilon_{1}\right)$$

where *σ*_1_ and *σ*_2_ are in correspondence with the direction of the *x*-axis and *y*-axis, respectively, as shown in Figure 3c. A and B are calibration coefficients obtained by the calibration samples [20], as shown in Figure 4.

**Figure 5:**
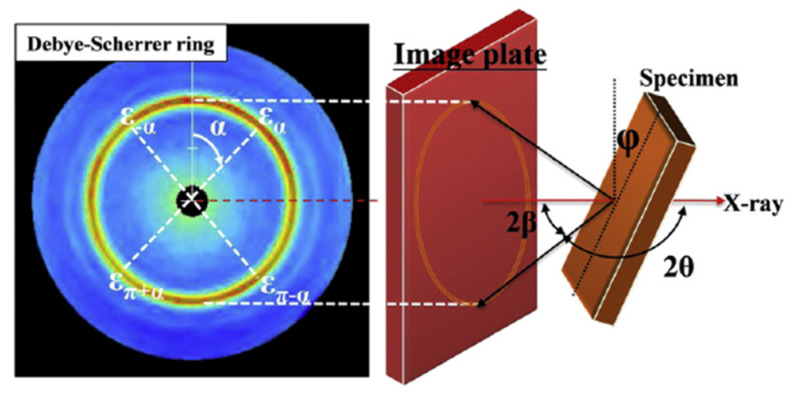
A schematic representation of the measurement of the Debye–Scherrer ring and the four types of strains used for stress calculation based on the cos*α* method [1].

The XRD method is widely applied to determine RS in samples without damage. The most recent XRD technique is the cos*α* method, which employs a 2D plane X-ray instead of the point or line in the sin^2^Ψ method, making it more effective and accurate. As shown in Figure 5, the RS *σ*_x_ in the x-direction is:

(3) $$\varepsilon_{\alpha 1}=\frac{1}{2}\left[\left(\varepsilon_{\alpha }-\varepsilon_{\pi +\alpha }\right)+\left(\varepsilon_{-\alpha }-\varepsilon_{\pi -\alpha }\right)\right]$$

(4) $$\sigma_{x}=-\frac{E}{1+v}\frac{1}{\sin2\beta }\frac{1}{\sin2\varphi }\frac{\partial \varepsilon_{\alpha 1}}{\partial \cos\alpha }$$

where *E* is Young’s modulus, *ν* is Poisson’s ratio, and *α*, *β* and *φ* are the angles shown in Figure 5. Additionally, *ε_α_*, *ε_π+α_*, *ε_−α_* and *ε_π−α_* are the strains obtained from diffraction beams in corresponding angles in Figure 5, where *ε_α_*_1_ can be calculated according to Equation (3), and *σ_x_* can be obtained via Equation (4).

**Figure 6:**
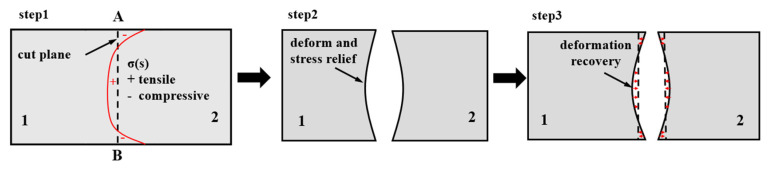
A schematic representation of the principle and steps of the contour method.

The contour method is a relatively new measurement method that can measure the inner RS of the sample [21]. The procedures are simpler and require fewer instruments compared with the deep hole drilling(DHD) method [22] and the deep hole contour (DHC) method [23]; moreover, the cut plane 2D-RS could be exhibited, and the cut half-samples were suitable for the comparison of mechanical properties in quenching and aging conditions in Section 2.1. The principle and steps are: (1) Cutting the sample into two same halves along the AB direction in Figure 2. (2) Releasing the RS perpendicular to the direction of the cross-section with no more constraint on the new surface, and the deformation of the cross-section after cutting is measured by the coordinate measurement machine HEXAGON GLOBAL 575 at a constant distance of 3 mm along the *x*-axis and 1 mm along the *z*-axis. (3) The measured deformation was data processing first and then applied in the elastic finite element model for the inverse calculation (the red arrows in Figure 6), which provided that the deformation recovered by an external force equal to RS in numerical value (red curve in Figure 6), whose direction was normal to the cut plane before cutting, as illustrated in Figure 6.

### 2.3. Microstructure Characterizations

Specimens for electron backscatter diffraction (EBSD) measurement were prepared by argon ion polishing at 4 KV for 4 h, the EBSD measurement for grain morphology and texture was tested on Gemini300 with an Oxford C-nano probe, and scanning electron microscopy (SEM) analysis for fracture appearance of tensile specimens was carried out on ZeissSigma 300.

## 3. Numerical Simulation

Material parameters experiments are mainly applied for numerical simulation with the finite element method (FEM). Material parameters of density, thermal conductivity, expansion coefficient, Yong’s modulus, Poisson’s ratio, etc., at different temperatures were tested, respectively. The heat transfer coefficient between the surface of the sample with 20 °C and 100 °C water or air was calculated by the lumped heat capacity method (LHCM) [24], with a dimension of 120 mm × 120 mm × 40 mm. A hot compression test with a reduction of 20% was carried out at a temperature of 100–500 °C at an interval of 50 °C and room temperature, and strain rate of 0.001 s^−1^ to obtain the stress-strain relationship.

A one-eighth-size model was used to analyze RS in quenching treatment. The coupled temp-displacement analysis model was used in FEM software ABAQUS. The elastoplastic model and Huber–Mises–Hencky (H–M–H) yield criterion were applied.

## 4. Results and Discussion

### 4.1. Microstructure

**Figure 7:**
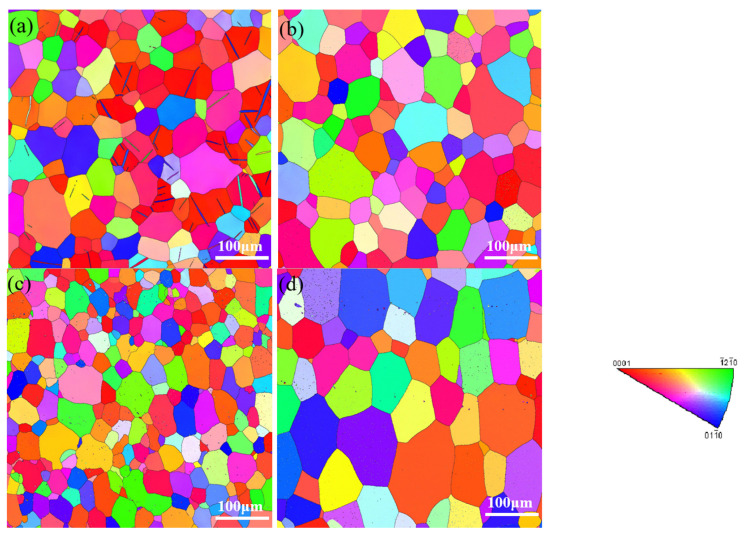
Grain information by EBSD in (**a**) WQ (20 °C), (**b**) WQ (100 °C), (**c**) AC and (**d**) WQ (100 °C) with aging.

WQ (20 °C), WQ (100 °C), AC and WQ (100 °C) with aging conditions were shown in the EBSD graphs in Figure 7a–d, respectively. Through the linear intercept method, the grain size of WQ (20 °C), WQ (100 °C) and AC conditions were 37 μm, 41 μm and 33 μm, respectively; there was no significant difference in grain size among these conditions, while that of WQ (100 °C) with aging afterward was 46 μm, with a growing trend. The twinning effect was observed in WQ (20 °C), which could be explained by the deformation caused by the large temperature gradient in the quenching process, while the phenomenon disappeared in WQ (100 °C) and AC, indicating the deformations were too small to activate twinning [25].

In WQ (20 °C) and WQ (100 °C), precipitates were none or few; however, there were large amounts of precipitates in AC, exsolution before aging in AC would reduce the response to aging hardening, which is detrimental to improving mechanical strength through heat treatment.

Compared Figure 7b with Figure 7d, the grain size increased a little, while the precipitates also increased and grew. This was in consistent with the view that precipitation strengthening played a critical role in improving the strength [26].

The texture is a kind of orientation distribution, which means that the orientation distribution state of polycrystal can deviate from the random distribution state obviously, showing certain regularity. The plastic deformation during quenching had an influence on texture, and texture-induced softening would lead to the reduction of RS [27].

**Figure 8:**
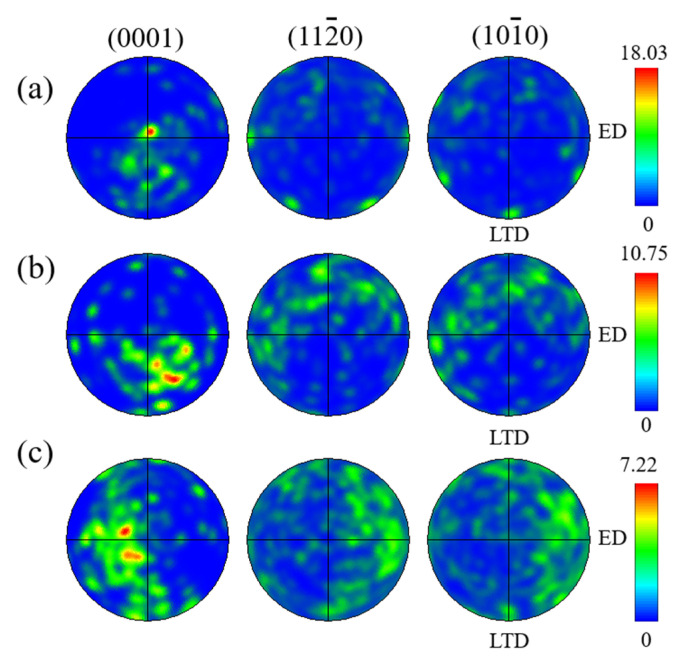
Pole figures of (**a**) WQ (20 °C) (**b**) WQ (100 °C) (**c**) AC.

To manifest the relationship between texture intensity and residual stress of WQ (20 °C), WQ (100 °C) and AC, pole figures are shown in Figure 8, which are widely used to describe the texture intensity, the max values are 18.03, 10.75 and 7.22, respectively, the order of the values were as follows: WQ (20 °C) > WQ (100 °C) > AC. This could be explained by the large quenching rate and temperature gradient, which brought about large plastic deformation and RS, and the large deformation increases the texture intensity. It is consistent with the phenomenon that the RS decreased and the texture weakened as well in the homogenization process prior to extrusion of ME21 magnesium alloy extruded plates [28].

### 4.2. Residual Stress

**Figure 9:**
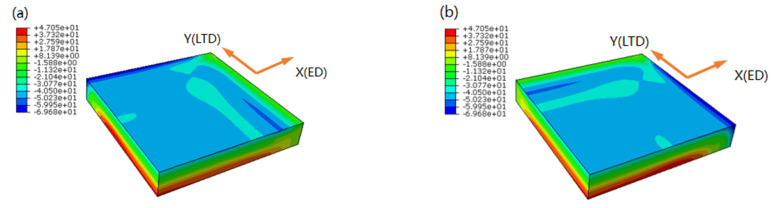

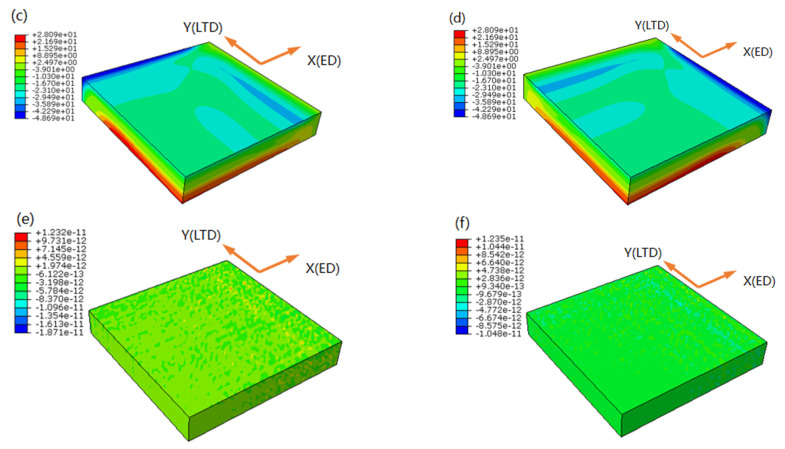
Simulation results of the distribution of (**a**) *σ_x_* in WQ (20 °C), (**b**) *σ_y_* in WQ (20 °C), (**c**) *σ_x_* in WQ (100 °C), (**d**) *σ_y_* in WQ (100 °C), (**e**) *σ_x_* in AC and (**f**) *σ_y_* in AC.

The simulation results of quenching RS are shown in Figure 9, only *σ_x_* and *σ_y_* are presented as *σ_z_* is close to zero in all cases because the samples are comparatively thin. *σ_x_* and *σ_y_* are absolutely the same in value and symmetry in distribution by comparing (a,c) with (b,d), because the length in the *x* and *y* direction of the sample is equivalent, and the yield criterion is isotropic. *σ_x_* and *σ_y_* in (e,f) are virtually zero. The RS generated by water quenching is compressive on the surface and tensile in the center [29], and has a layered distribution along the thickness direction of the sample, only near the edge there is a compressive stress concentration. The RS is mainly due to temperature gradient and inhomogeneous plastic deformation, at the beginning of quenching, the sample surface exchanged heat with the surrounding water and cooled rapidly, then the internal heat was transferred to the surface and exchanged heat with the surrounding water, because the surface cooled faster than the center, in addition, the heat exchange rate with water was much larger than heat transfer rate in the sample, a large temperature gradient was formed, which led to nonuniform thermal strain between surface and center, when the sample cooled down, RS was formed.

RS was ranging from −69.68 MPa on the surface to 47.05 MPa in the center in WQ (20 °C), ranging from −48.69 MPa on the surface to 28.09 MPa in the center in WQ (100 °C) from a to d, and almost zero from e,f. RS in WQ (100 °C) has reduced by ~30% on the surface and ~40% in the center compared with in WQ (20 °C).

**Figure 10:**
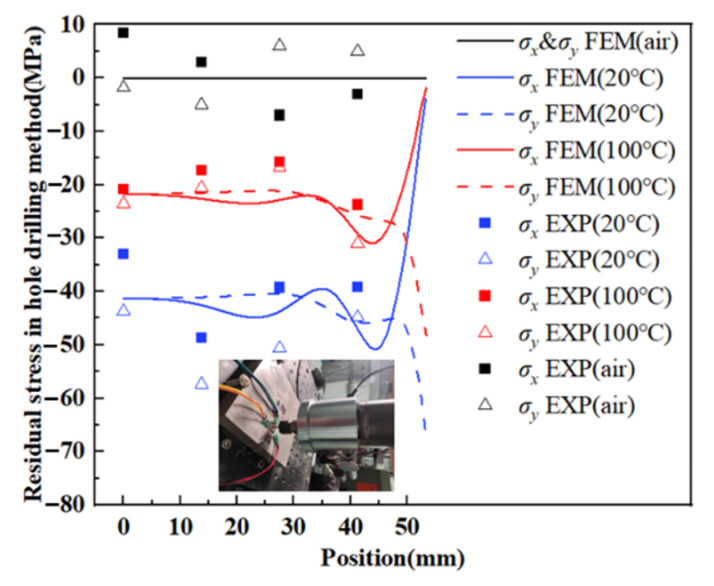
Simulation and experimental results of RS in hole drilling method along path AB.

**Figure 11:**
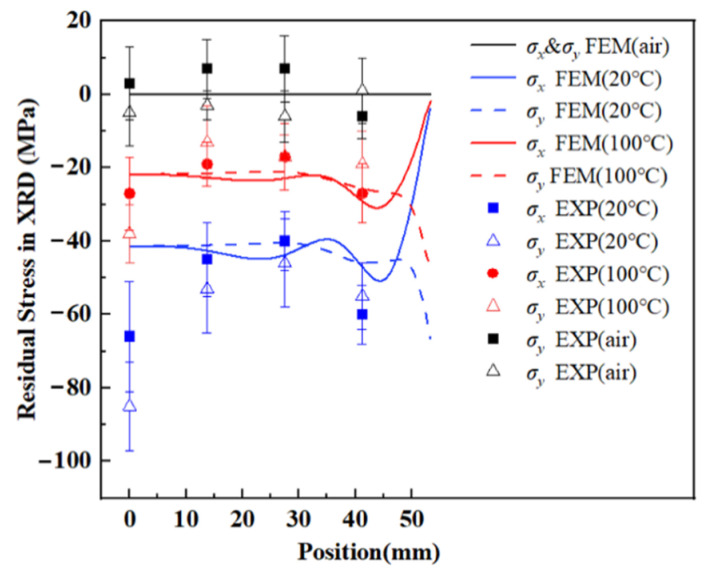
Simulation and experimental results of RS in XRD along path AB.

For a more quantitative understanding, simulation results of RS in the samples in the path from A to B were extracted in Figure 10 and Figure 11. The curves of RS in WQ (20 °C) and WQ (100 °C) have the same trend, namely, in more than half of the dimensions (0–35 mm), the curves were flat with fluctuations less than 5 MPa, RS in WQ (100 °C) vary from −25 to −20 MPa, and in WQ (20 °C) vary from −45 to −40 MPa. However, the absolute value of *σ_x_* declined to zero and *σ_y_* rose to its maximum fiercer in 35–55 mm. This is because the surface near the middle can only transfer heat with water in the Z direction, while the surface near the edge can transfer heat both in the X and Z directions, so the heat exchange is larger, resulting in a more drastic variation in RS. Moreover, RS in AC was so tiny that further analysis was unnecessary. Experimental results of RS, both in the hole drilling method and the XRD method, were also plotted in Figure 10 and Figure 11.

The points of the experimental values were distributed near the curves of the simulation results, most of their errors were within ±10 MPa both in the hole drilling method and the XRD method, the absolute value of experiment values of RS obeyed the following orders: WQ (20 °C) > WQ (100 °C) > AC, which were in consistent with the trend of simulated results. Furthermore, the test results were various between the two methods even at the same point as the measured depth of RS is different [19].

**Figure 12:**
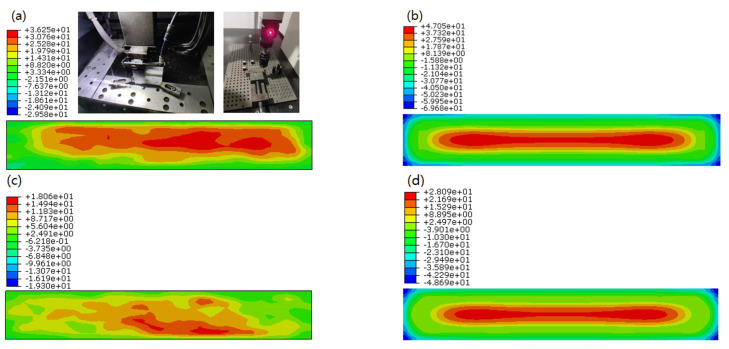
Inner RS of (**a**) experimental results in WQ (20 °C), (**b**) simulation results in WQ (20 °C), (**c**) experimental results in WQ (100 °C) and (**d**) simulation results in WQ (100 °C) in contour method.

The inner RS of the samples measured by the contour method were shown in Figure 12a in WQ (20 °C) and Figure 12c in WQ (100 °C), Figure 12b,d were corresponding simulation results. The edge of the cross-sections could not be measured with the coordinate measurement machine; therefore, the data processing was not accurate due to outward interpolation; only RS within the range of measurement points in Section 2.2 was considered, so the tensile RS in the center part of the cross-sections was more accurate than compressive RS in the edge part of the cross-sections in Figure 12, and tensile RS was the main topic discussed below.

From Figure 12b,d, it is revealed that RS distribution in the cross-sections was totally symmetry from the edge to the center, and it was a “runway” type distribution theoretically, with compressive outside and tensile inside, the only difference was the maximal tensile RS value in WQ (20 °C) and WQ (100 °C) were 47.05 MPa and 28.09 MPa, respectively. However, compared with Figure 11 the RS distribution was still the largest tensile stress in the middle area of the cross-sections, but it is not regular. The main reason was that the boundary conditions during quenching are not as symmetric as the finite element simulation. For example, the generation of bubbles has certain randomness, and the generation and breakage of bubbles are inconsistent on the upper and lower surfaces.

**Figure 13:**
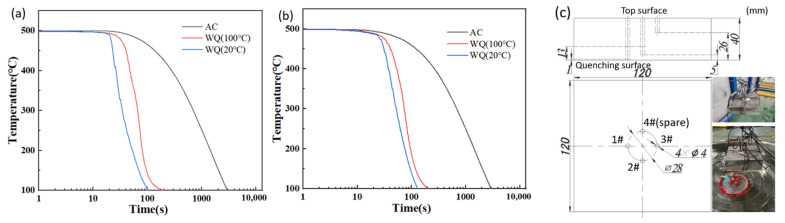
Measured cooling curves in AC, WQ (100 °C) and WQ (20 °C) (**a**) in the 1 mm deep (1#) (**b**) middle part (3#) of the sample by the lumped heat capacity method (**c**) sample of heat transfer coefficient experiment.

To quantify the inhomogeneity of temperature decrease which induced RS, cooling curves of the thermocouple at 1 mm away from the bottom surface (1#) and in the middle part of the sample (3#) by lumped heat capacity method (together with 2# at 5 mm away from the bottom surface) in WQ (20 °C), WQ (100 °C) and AC were shown in Figure 13.

The overall trend of temperature decrease was slow at first, then fast, and then slowed again. The calculation of the cooling rate was to take the approximate linear segment of 450–150 °C temperature difference (=300 °C) and then divided it by the corresponding cooling time under different cooling conditions, as shown in Table 1. Under the same cooling conditions, there is a temperature difference between the near-surface and the middle part because of the different cooling rates, which could be listed in the following orders: WQ (20 °C) > WQ (100 °C) > AC, so was the magnitude relationship of RS under these conditions.

Nonetheless, the errors between simulation and experimental results were not small enough both on the surface and in the center, for example, in WQ (20 °C) at A point, experimental *σ_x_* and *σ_y_* in the hole drilling method were −32.96 MPa and −43.70 MPa, respectively, while the corresponding simulation value was −41.35 MPa, the maximal experimental inner RS in WQ (20 °C) was 36.25 MPa, while the corresponding simulation value was 47.05 MPa; this should mainly attribute to the flowing reasons:

There existed inevitable measurement errors in the experiments of material parameters varying with temperature. Take constitution equation and heat transfer coefficient as examples, temperature, strain rate and strain are the major factors affecting the stress-strain relationship, while the hot compression test didn’t take strain history into account [29]. Heat transfer coefficient was obtained by lumped heat capacity method under the assumption of a one-dimensional heat conduction model through a single-side quenching experiment, but the size of the experimental sample was restricted, the side and the top surface would exchange heat with air inevitably, furthermore, the experimental condition of single-side quenching under one-dimensional heat conduction model was not completely consistent with that of actual quenching process due to bubble floating on the side and the top surface, which above would bring about a certain error in simulation. On the other hand, the elastoplastic model and H–M–H yield criterion assume that the materials are continuous, homogeneous and isotropic, while grain size would change and texture may vary actually, making the actual situations and theoretical models different.

### 4.3. Mechanical Properties

**Figure 14:**
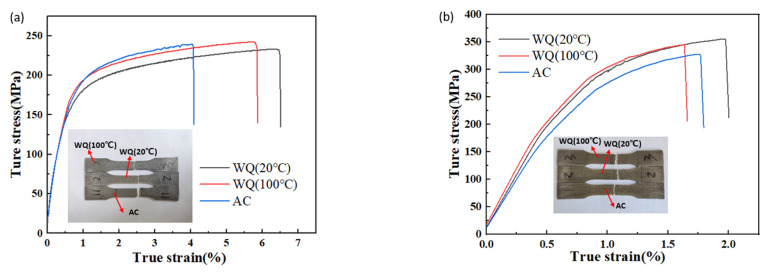
Stress–strain curves and fractured location of the tensile specimens in (**a**) quenching state and (**b**) aging state.

One of the tensile samples under each working condition is selected, and the stress-strain curves were depicted in Figure 14. In quenching state, the stress increases rapidly with the increase of strain at first, which is due to the increase of dislocation density caused by interactions and multiplications [30], then it became stable until fractured, while in aging state, this phenomenon was not obvious for early fracture. From the fractured position of the curves, orders of ductility in quenching state were as follows: AC < WQ (100 °C) < WQ (20 °C), while it was close in aging state, ranging from 1.6% to 1.9%.

**Figure 15:**
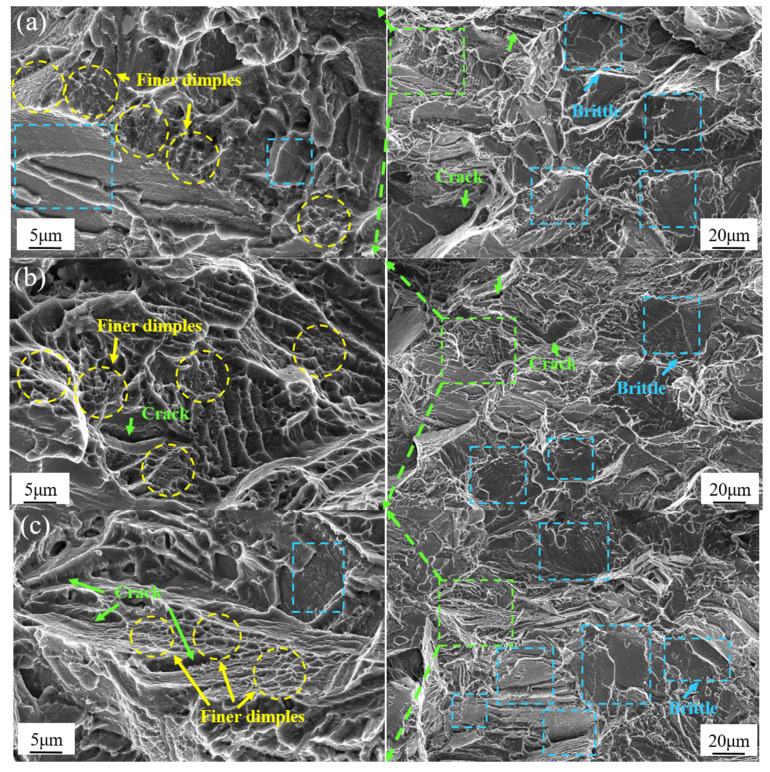

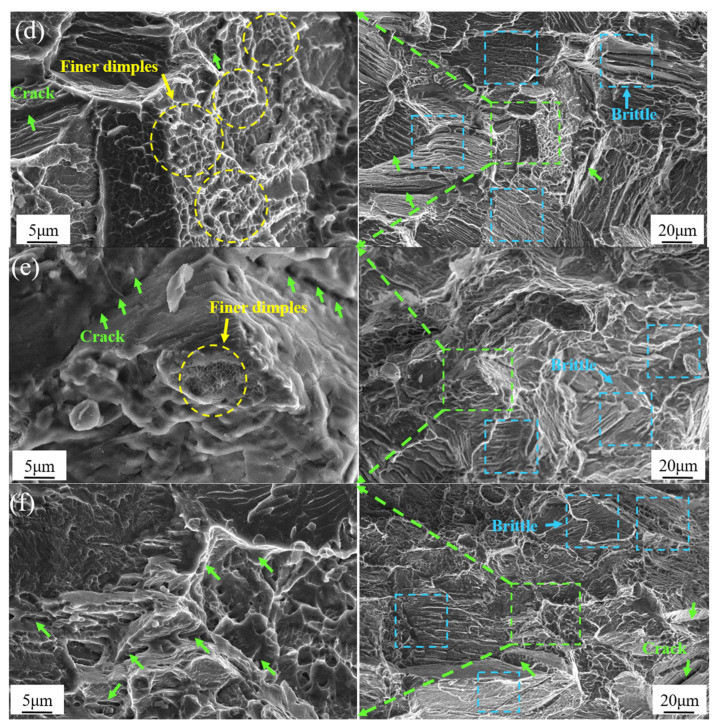
SEM images of tensile specimens in (**a**) WQ (20 °C), (**b**) WQ (100 °C), (**c**) AC, (**d**) WQ (20 °C) with aging, (**e**) WQ (100 °C) with aging and (**f**) AC with aging states.

The fracture modes are mainly divided into dimple and cleavage fractures, and whether the fracture type is ductile or brittle depends on the plastic strain of the material before fracture [31]. As illustrated in Figure 14, the ductility of this alloy was lower than that of the magnesium alloy with LPSO phase [32] and Mg-5Zn-3.5Sn-1Mn-0.5Ea-0.5Cu alloy [29], brittle fracture may probably happen in this case. For more detailed information, SEM images were shown in Figure 15 to explore the microscopic behavior of fracture.

In all the figures, brittle fractures and cracks commonly exist, no matter in what conditions, dimple fractures representing ductility were seldom observed, which could explain the poor ductility of the specimens. In quenching conditions, brittle fractures in Figure 15c were more than the other two in Figure 15a,b, which could prove that ductility in AC was the lowest in quenching states. As the magnification increases, finer dimples were also found in quenching conditions, while in aging states, finer dimples could be found in Figure 15d and few or no finer dimples exist in Figure 15e,f, which proved that ductility in quenching state was better than that in aging state, and reveals that high quenching rates improve ductility to a certain extent [32].

**Figure 16:**
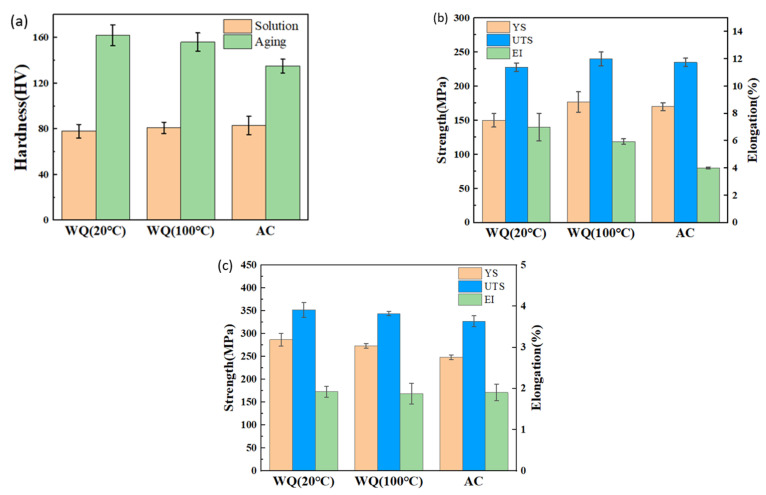
(**a**) Hardness in quenching and aging state (**b**) strength in quenching state (**c**) strength in aging state.

The hardness in quenching and aging states was shown in Figure 16a, they were 78 Hv, 81 Hv and 83 Hv in WQ (20 °C), WQ (100 °C) and AC, respectively, for quenching after solution state. However, the corresponding values were 162 Hv, 156 Hv and 135 Hv. The strength of the alloy was consistent with the hardness in quenching and aging states, as illustrated in Figure 16b,c; WQ (100 °C) has the highest yield strength (YS) and ultimate tensile strength (UTS) in the quenching state, while after aging the relationship could be listed in the following orders: WQ (20 °C) > WQ (100 °C) > AC. The enhancement of YS in WQ (20 °C), WQ (100 °C) and AC were 137 MPa, 96 MPa and 78 MPa, respectively, and that of UTS were 124 MPa, 104 MPa and 92 MPa, respectively. The elongation (EI) varied from 4 to 7% in the quenching state and decreased to 1.87–2% after aging.

The enhancement of strength could be explained by precipitation and hardening during aging. The Mg-Gd-Y alloys exhibit a remarkable age-hardening response due to the fine lenticular-shaped *β*’ precipitates, which can impede dislocation slip effectively [3]. Rapid quenching maintains a supersaturated solid solution state, and while the cooling rate gets lower, coarser precipitates appear before aging [29,32], the phenomenon has a negative influence on aging hardening.

Time–temperature–property (TTP) and time–temperature–transformation (TTT) curves are utilized to evaluate quenching sensitivity for aluminum alloys [33]. Taking that 95% of the maximum hardness and the critical temperature range varies from 300 to 400 °C as a given, the corresponding transformation time for 2A14 aluminum alloy is 10 s [34]. However, the TTP and TTT curves are probably not suitable for magnesium alloys as the diffusion rate of elements in magnesium alloys is considerably slower, and the quenching sensitivity parameter *Q* values [26,32] are much less compared with that of aluminum alloys [35]; hence, 95% of the maximum hardness and strength [36] criterion rather than time could be a better option to evaluate whether the mechanical properties are qualified.

**Figure 17:**
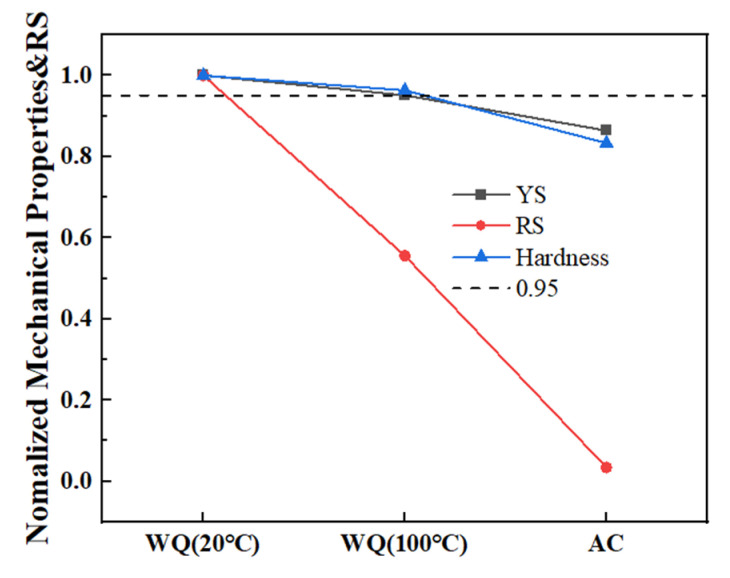
Normalized mechanical properties and RS.

The normalized YS, RS and hardness were depicted in Figure 17 to make a quantitative comparison. *σ_y_* of the point A declined greatly to 55.6% in WQ (100 °C) and 3.4% in AC, while less sensitive as to YS and hardness, with only 4.9% and 3.7% loss on YS and hardness in WQ (100 °C), with 13.6% and 16.7% loss on YS and hardness in AC. Nevertheless, the strength loss in AC was still unacceptable according to the view that the strength should remain no less than 95% of the highest value. So, WQ (100 °C) was the best quenching condition following solution treatment in this study, and it comes to a conclusion that it is feasible to invent an appropriate quenching method of greatly reducing RS while maintaining mechanical properties. The results would be beneficial to the application of the alloy.

## 5. Conclusions

The effect of quenching cooling rate on microstructure, RS and mechanical properties of a rare-earth wrought magnesium alloy Mg-Gd-Y-Zr-Ag-Er was investigated in the methods of 20 °C water quenching (WQ (20 °C)), 100 °C water quenching (WQ (100 °C)) or air cooling (AC), and the results revealed that it is possible to invent an appropriate quenching method of greatly reducing RS while maintaining mechanical properties. The main conclusions were drawn as follows:

1. The decrease of quenching rate has little effect on the grain size, but makes the twinning disappear, precipitates increase and the texture weakened, leading to easier brittle fracture after aging;
2. WQ (100 °C) has the highest yield strength (YS) and ultimate tensile strength (UTS) in the quenching state. Cooling rates, RS and mechanical properties after aging could be listed in the following orders: WQ (20 °C) > WQ (100 °C) > AC;
3. The quenching RS declines greatly in WQ (100 °C) and close to zero in AC compared with WQ (20 °C) as cooling rates decrease, while YS and hardness only decrease by 4.9% and 3.7% in WQ (100 °C), 13.6% and 16.7% in AC after aging;
4. WQ (100 °C) is the best quenching condition following solution treatment in this study, and it is feasible to greatly reduce RS while maintaining mechanical properties. The results would be beneficial to the application of the alloy.

## Figures and Tables

**Table 1:** Cooling rates in AC, WQ (20 °C) and WQ (100 °C).

Cooling Rates	1 mm Deep (1#) (°C/s)	Middle Part (3#) (°C/s)
WQ (20 °C)	6.38	5.41
WQ (100 °C)	5.36	4.67
AC	0.16	0.14

## Data Availability

The data presented in this study are available on request from the corresponding author.
